# A pilot study into the effects of PTSD-assistance dogs’ work on their salivary cortisol levels and their handlers’ Quality of life

**DOI:** 10.1080/10888705.2023.2259795

**Published:** 2023-09-19

**Authors:** Karoline Gerwisch, Karl Weissenbacher, Michelle Proyer, Rupert Palme, Ludwig Huber

**Affiliations:** a Comparative Cognition, Messerli Research Institute, University of Veterinary Medicine Vienna, Medical University of Vienna and University of Vienna, Vienna, Austria; b Testing and Coordination Centre for Assistance Dogs, Therapy Companion Dogs and Animal Welfare Qualified Dog Trainers, Messerli Research Institute, University of Veterinary Medicine Vienna, Vienna, Austria; c Department of Education, University of Vienna, Vienna, Austria; d Department of Biological Sciences and Pathobiology, University of Veterinary Medicine, Austria

**Keywords:** Assistance dog, PTSD, quality of life, saliva, cortisol

## Abstract

Assistance dogs for people with Posttraumatic Stress Disorder (PTSD) support their handlers by performing tasks that are supposed to mitigate the effects of their mental disability. This study examined the Quality of Life (QoL) of PTSD-assistance dogs’ handlers in Austria and Germany using a qualitative online questionnaire based on the Capability Approach. To correspondingly explore whether the involved assistance dogs experience distress triggered by their daily schedules, we measured their salivary cortisol values. These were compared to the cortisol levels of companion dogs without special tasks, as well as diabetic-signal dogs that have a similar workload. Our results showed that people suffering from PTSD-symptoms can improve their QoL with the aid of their assistance dog. However, being accompanied by an assistance dog creates new social barriers. Surprisingly, we found significantly lower salivary cortisol levels in PTSD-assistance dogs compared to the control groups. We conclude that a positive relationship between PTSD-assistance dogs and their handlers can reduce stress on both sides, and that training well tuned to the requirements of an assistance dog can prevent stress in their daily lives.

## Introduction

Nowadays, as people are affected by the COVID-19 pandemic but also by war, forced migration and other crises, dealing with trauma and related PTSD seems to be particularly important. The reality of directly affected people’s lives, and also of those around them, can be forever altered by such drastic traumatizing events. PTSD is an anxiety disorder that can appear after exposure to these life-threatening events or traumas. The complex chronic stress phenomenon is linked to symptoms of intrusive thoughts, avoidance or numbing of emotions, dissociation (Glenk & Kothgassner, 2017), reexperiencing traumatic events including flashbacks or nightmares, and hyperarousal, which can be manifested through difficulty sleeping and concentrating (Institute of Medicine of the National Academies, 2006). PTSD-assistance dogs are trained to reduce the impact of specific symptoms for people living with this condition and might help to improve their overall QoL. In the current study, we refer to QoL as not only the beings and doings of an individual and their opportunities to realize them but also the personal perception of their position in life in relation to goals, expectations, standards and concerns. This includes the individual’s own psychological health, comfort and participation in life events (Jenkinson, 2023; Robeyns & Fibieger Byskov, 2021; World Health Organization, 2012). Beings and doings are defined as people’s genuine opportunities, for example, to move around freely or have supportive social relationships (Robeyns & Fibieger Byskov, 2021). Examples of the assistance dogs’ tasks are to give PTSD-patients a sense of safety, help to improve interpersonal connections, encourage engagement in the community, and regain areas of functioning that may have been diminished by the individual’s trauma (Assistance Dogs Australia, 2022).

Little is known about the level of impact which PTSD-assistance dogs actually have on the QoL of their handlers, except in the area of American war veterans. One preliminary study has shown lower overall PTSD-symptoms of veterans with assistance dogs compared to those without (O’Haire & Rodriguez, 2018). Moreover, research on the welfare of the PTSD-assistance dogs themselves is severely lacking. Although measurements of dogs’ salivary cortisol levels have been carried out in previous studies with search and rescue dogs as well as companion and shelter dogs (Cobb, Iskandarani, Chinchilli, & Dreschel, 2016; Wojtaś, Karpiński, & Czyżowski, 2020), such studies have so far not been conducted on PTSD-assistance dogs. However, a study conducted with dogs in Animal Assisted Therapy (AAT) and Animal Assisted Activities (AAA) showed significantly higher salivary cortisol levels on therapy days than on control days (Haubenhofer & Kirchengast, 2007). In contrast, a comparison of salivary cortisol levels of diabetic-signal dogs, therapy dogs and companion dogs showed no significant differences (Bruckner, 2019).

Based on the findings of the mentioned study which showed that PTSD-assistance dogs have a positive impact on the QoL of war veterans, we hypothesized that PTSD-assistance dogs would also affect PTSD-sufferers’ condition in a positive way. Therefore, the first aim of this study was to examine how assistance dogs affect the lives of PTSD-patients. To do so, we assessed the QoL of the participants by developing a qualitative online questionnaire based on Martha Nussbaum’s central human capabilities (Nussbaum, 2006; Nussbaum, Sen, & World Institute for Development Economics Research (Eds.), 1993) and quantitative standardized instruments, i.e., objective, non-disease-specific questionnaires, calculating patients’ health state (Devlin & Brooks, 2017).

The second aim of the study was to understand whether PTSD-assistance dogs face more stressful situations during their daily work compared to control groups consisting of companion dogs and diabetic-signal dogs. This research is relevant for the improvement and maintenance of assistance dogs’ welfare, since distress can impair psychological, physiological, immunological, and behavioral functions (Glenk & Kothgassner, 2017). Previous studies have shown varying results regarding the stress levels of assistance and therapy dogs. Higher cortisol levels have been found in dogs during their work in Animal Assisted Interventions (AAI). We, therefore, hypothesized that the salivary cortisol levels of PTSD-assistance dogs are higher than those of companion dogs without special tasks, due to working stress, like being permanently alert and coping with patients’ flashbacks. To measure this, we asked the human participants to collect saliva samples of their assistance dogs, so we could measure cortisol values as biomarkers of chronic stress. Finally, we compared these data with those sampled earlier from dogs of two control groups, companion dogs without special tasks and diabetic-signal dogs.

## Material and methods

### Subjects

In the first part of our study, people with PTSD (*N* = 24) who currently have an assistance dog participated in an online questionnaire to assess their QoL. For the measurement of cortisol levels, saliva samples were collected from the participants’ PTSD-assistance dogs (*N* = 9), who were certified according to § 39a of the federal disability act and registered by the Testing and Coordination Centre for Assistance Dogs, Therapy Companion Dogs and Animal Welfare Qualified Dog Trainers at the Messerli Research Institute, University of Veterinary Medicine Vienna. The sample consisted of nine neutered dogs (Labrador Retrievers, mixed breeds, Australian Shepherd and Cavalier King Charles Spaniel) − 5 males and 4 females (age mean 4.1 years).

Data of companion dogs (*N* = 8, 1 male and 7 females, age mean 4.4 years) and certified diabetic-signal dogs (*N* = 9, 5 males and 4 females, 3.5 years) were used from a previous study (Bruckner, 2019) for comparison. The control groups’ characteristics were similar to our sample of PTSD-assistance dogs, as they were neutered, retriever-like dogs of both sexes (see Table 1). We chose diabetic-signal dogs as an assistance dog control group since they have a similar workload to our sample. Both assistance dog groups must be ready for action in case of PTSD symptoms (flashbacks, seizures, etc.) or hyper-/hypoglycemia.

### Measurements

Data concerning the QoL of people with PTSD in relation to their assistance dogs was gathered via a qualitative approach, since qualitative data can provide a more detailed understanding of a problem by including personal experiences of the focal groups’ everyday life (Tedeschi & Jenkins, 2019). In contrast, a quantitative approach was used for the comparison of cortisol levels of the three groups of dogs. Both approaches offer different perspectives but provide a more complete understanding of the research subject when combined (Creswell, 2017).

### QoL measure

We assessed QoL using a qualitative online questionnaire (https://www.soscisurvey.de/). The questionnaire had open questions to give participants the opportunity to describe their individual points of view and personal experiences (see supplementary material S1). Participants were also asked to select all statements about life with an assistance dog which were applicable to them in a presented table.

In her Capability Approach, Nussbaum (2006) proposed 10 capabilities as central requirements for a life with dignity, which can be further adapted and specified by any given society. We used five capabilities (life; bodily integrity; senses, imagination and thought; emotions; affiliation) as the basis of the questionnaire, since those are affected by the changes of functioning through PTSD. Further, we used standardized instruments to assess health-related QoL, namely EQ-5D-5 L (Devlin & Brooks, 2017; EuroQol Research Foundation, 2021) and WHOQOL-BREF (World Health Organization, 2012).

Participants received an invitation link to the online questionnaire and completed it in their home environment to make them feel comfortable and avoid any stress or flashbacks. Participants were also offered the possibility to pause the process of filling in their answers and come back to it at another time whenever they needed a break.

We then conducted a Qualitative Content Analysis, a standard evaluation method of qualitative, empirical research (Mayring, 2015). It is particularly useful to systematically describe the meaning of rich data requiring interpretation (Schreier, 2012) and to focus on the subjects and their individual experiences. For the coding process, we used the software MAXQDA (https://www.maxqda.com/de/).

### Salivary cortisol measure

Ten PTSD-assistance dog handlers of the sample who participated in the questionnaire volunteered to take saliva samples of their dogs with a cotton roller (Cortisol-Salivette^®^, SARSTEDT AG & Co, Germany) in the same way as in several studies before (Bruckner, 2019; Glenk et al., 2013, 2014; Haubenhofer, Möstl, & Kirchengast, 2005; Kobelt, Hemsworth, Barnett, & Butler, 2003). Saliva sampling was executed by the study participants in their home environment to avoid external stresses for the dog, such as from being handled by an unfamiliar person in an unknown environment (Cobb, Iskandarani, Chinchilli, & Dreschel, 2016; Jones et al., 2014). The handlers were asked to take these samples three times a day (morning – noon – evening) on seven consecutive days. The time of sampling varied to some extent due to their different daily routines.

Analyses of the samples for cortisol content were performed at the Institute of Biochemistry of the University of Veterinary Medicine Vienna by using a highly sensitive cortisol enzyme immunoassay (EIA) (Palme & Möstl, 1997), which has been previously used in dogs’ stress assessments (Bruckner, 2019; Glenk et al., 2014; Haubenhofer, Möstl, & Kirchengast, 2005).

### Statistical analysis

We tested the effect of dog type (with three levels: PTSD dog, companion dog and signal dog) by fitting a linear mixed model in R (version 4.1.2, R Core Team, 2021), using the function lmer from the lme4 package (version 1.1–27.1, Bates, Mächler, Bolker, & Walker, 2015). For the key-fixed effects of interest, we used dog type and time of day, as well as their interaction. As additional fixed effects that served as control variables, we included age and sex (levels: female and male). Before being included in the model, we z-transformed age and time of day to ease model convergence and achieve easier interpretable model coefficients (Schielzeth, 2010). We inspected the response to see whether its distribution was roughly symmetric, and since this was not the case, we log-transformed (base e) cortisol level. We excluded one observation because its cortisol value was much lower than what is expected to be measured with good confidence (value = 0.08). The way we avoided pseudoreplication, checked for the assumptions of normal distribution and homogeneous residuals, and we assessed model stability is described in the Supplementary Materials (S3).

We then bootstrapped the model estimates (to estimate confidence intervals) using the function bootMer of the package lme4. Next, we compared each full model with all terms included, to their respective null model lacking the key terms of interest but otherwise being identical in the random effects part, using a likelihood ratio test to avoid “cryptic multiple testing” (Forstmeier & Schielzeth, 2011). If the full-null model comparison revealed clear effects of the predictors of interest, we tested the individual fixed effects to achieve informative estimates of the fixed effects terms using the drop1 function in R. We did so by reducing model complexity and dropping non-significant interactions, from higher order to lower order terms, from the model one at a time and compare the simpler with the more complex model utilizing likelihood ratio tests.

We compared the estimated marginal means and used Tukey HSD post hoc tests, applied to the reduced model, to compare cortisol values among dog types. This was done using the emmeans function from the emmeans package (v.1.6.3, Lenth et al., 2023). Because we expected that state (e.g., working stress during an assistance task like calming the handler during a flashback) could also influence cortisol level, we also tested the effect of state separately from the other predictors using a similar linear mixed model. Only levels of state with at least 10 observations were included. Again, we compared estimated marginal means to compare cortisol values among different states.

### Ethical note

With regard to the human-related part of this study, the procedures have been discussed and approved by the ethics committee of the Medical University of Vienna in accordance with GSP guidelines, EK No. 1942/2021. The procedures of the dog-related testing have been discussed and approved by the institutional ethics and animal welfare committee in accordance with GSP guidelines and national legislation by the Ethics Committee of the University of Veterinary Medicine Vienna, Austria, ETK-187/12/2021.

## Results

### QoL measure

The study participants’ answers to the online questionnaire showed, that PTSD-assistance dogs affected their handlers’ lives by improving their health (e.g., by preventing injuries), giving them stability as well as responsibility and facilitating social contact. They also enabled a higher degree of mobility and provided their handlers with a sense of safety. In contrast, PTSD-assistance dog handlers got more unwanted attention than before they had their dogs and had to face new challenges, like access issues (see supplementary material S4 for further details).

### Salivary cortisol measure

The results of this study showed that the test predictors (dog type) had a clear impact on cortisol levels (full-null model comparison: χ^2^ = 26.108, df = 5, *P* < 0.0001). However, we found no significant interaction between dog type and time of day (interaction effect: χ^2^ = 0.29, df = 1, *P* = 0.866), therefore, we removed this interaction from the model.

**Figure 1:**
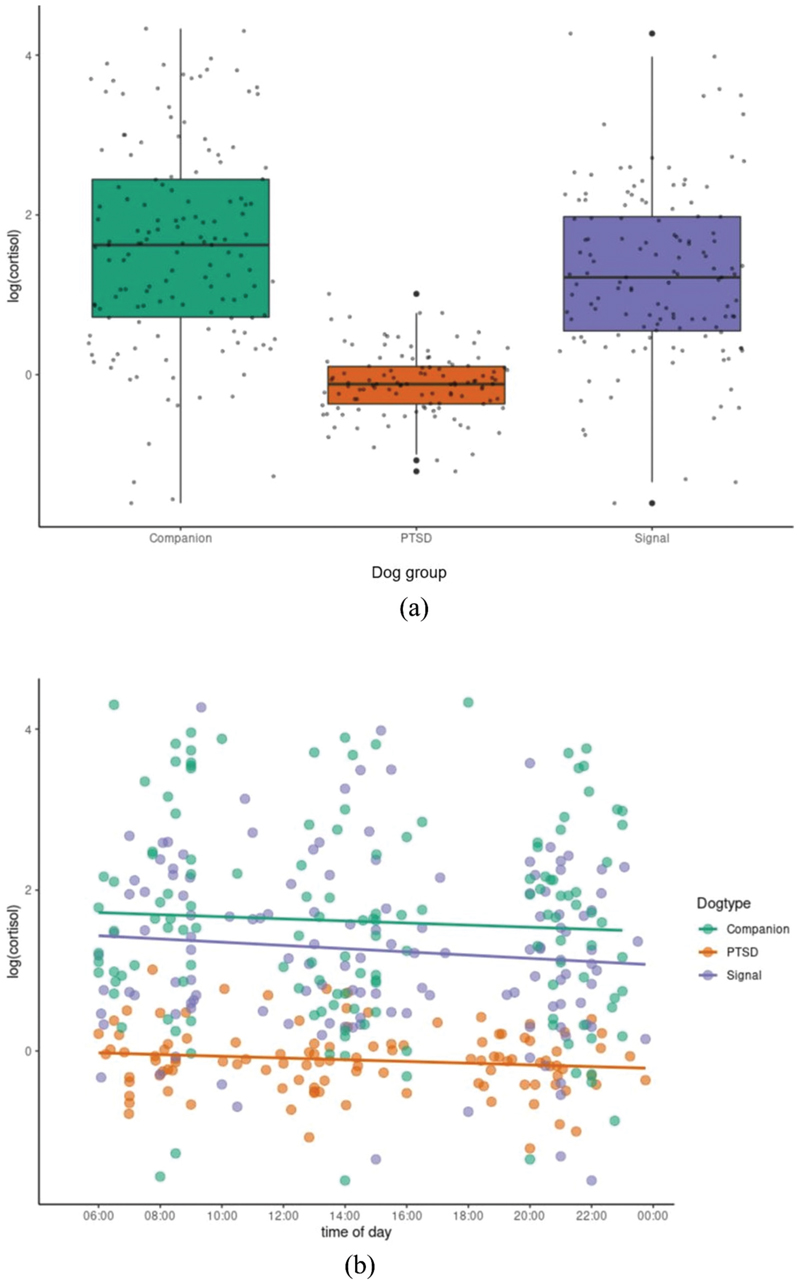
(a) cortisol levels of dog types. The box represvents the middle 50% of scores, upper and lower whiskers represent scores outside the middle 50%, circles represent outliers, and the black line represents the median. b) Saliva cortisol of dog types throughout the day. The dots represent values of data points, the three data clusters show the three sampling points of the day (morning, midday, evening). Trend lines indicate the correlational relationship between variables.

The reduced model, only including main effects, revealed that cortisol levels differed between dog types (χ^2^ = 24.19, df = 2, *P* < 0.0001). More specifically, PTSD-assistance dogs had significantly lower cortisol levels compared to the other dog types (Figure 1a) (PTSD-assistance dogs: $\tilde{\text{x}}=0.89$, s = 0.42, signal dogs: $\tilde{\text{x}}=3.38$, s = 9.35, companion dogs: $\tilde{\text{x}}=5.07$, s = 14.65). Mean values of salivary cortisol of the three dog groups reported on a non-logarithmic basis looked as follows: PTSD-assistance dogs: $\tilde{\text{x}}=0,96$ ng/ml, diabetic-signal dogs: $\tilde{\text{x}}=6,34$ ng/ml, companion dogs: $\tilde{\text{x}}=10,79$ ng/ml.

**Figure 2:**
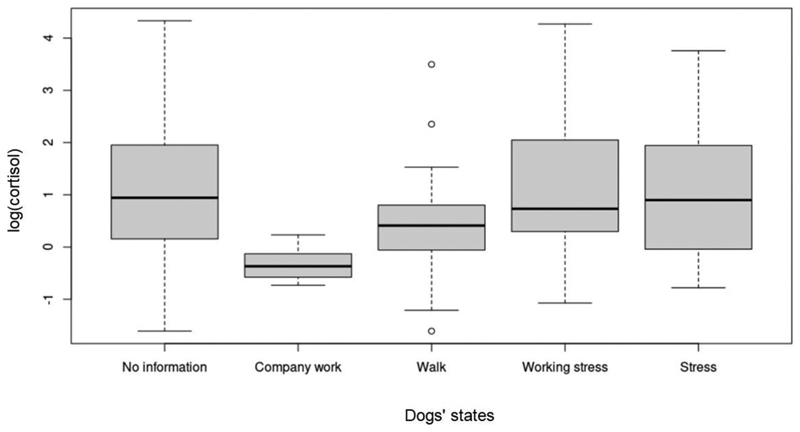
Saliva cortisol of dogs at different states before sampling. The features of the box plots are the same as in Figure 1a.

Time of day (Figure 1b), sex and age had no significant effects. There was also no significant effect of the PTSD-assistance dogs’ state (stress: $\tilde{\text{x}}=0.90$, s = 0.46, working stress: $\tilde{\text{x}}=0.56$, s = 0.28, company work: $\tilde{\text{x}}=0.69$, s = 0.22, walk: $\tilde{\text{x}}=0.93$, s = 0.49) (χ2 = 4.119, df = 4, *P* = 0.39) (Figure 2).

## Discussion

The findings of our study’s questionnaire provided evidence for the hypothesis that PTSD-patients experience a change in their everyday lives since they got their assistance dogs. We found that these dogs increase patient vitality and help them to gain access to higher autonomy in their everyday lives. Study participants seem to obtain an attachment figure from their dog, which in turn increases their social ability, improves (psychological) health and is a motivational driving force. However, aside from the positive impacts PTSD-assistance dogs have on the QoL, (social) challenges may also emerge from interacting with society and from keeping a dog.

Regarding the assistance dogs themselves, we found a significant difference of salivary cortisol values in PTSD-assistance dogs compared to companion dogs as well as diabetic-signal dogs. However, these results deviated from our predictions, with the cortisol levels of PTSD-assistance dogs being significantly lower than those of the control groups.

### QoL measure

The findings regarding the assistance work of interruption and signaling of dissociation by the focal dog group are in line with previous research on the effects of Human-Animal-Interactions during AAI, for example, for soldiers with PTSD (e.g., Beetz, Schöfmann, Girgensohn, Braas, & Ernst, 2019). HAI positively impact physiological parameters (e.g., cortisol levels) during stressful situations and physical contact between humans and animals is especially effective, since it increases the levels of the attachment hormone oxytocin, leading to mental and physiological relaxation (Beetz, Schöfmann, Girgensohn, Braas, & Ernst, 2019). The calming effect is even larger between humans and animals with a close relationship (Beetz & Bales, 2016), which applies to our study’s participants and their dogs. Moreover, focusing on the dog can have a distracting effect on PTSD-patients, retaining them in the here and now, and helping them to reduce their traumatic flashbacks and intrusive thoughts (Beetz, Schöfmann, Girgensohn, Braas, & Ernst, 2019).

Additionally, previous studies on veterans with PTSD living with dogs (O’Haire & Rodriguez, 2018; Rodriguez, Bryce, Granger, & O’Haire, 2018; Stern et al., 2013) reported a higher cortisol awakening response, which could be interpreted as an indicator of better health and well-being. In the present study, we also found better overall reported health of the participants related to having an assistance dog, reinforcing the findings of a higher vitality and QoL. Regarding the health of PTSD-patients related to their sleeping quality, they reported an improvement since the time they obtained an assistance dog, while contrary to that, the dog does not aid in sleeping through the night. The improved sleeping quality contributes to a better overall health of people with PTSD, since troubled sleep and chronic stress can impair humans’ health, as prolonged high cortisol levels have been shown to promote diseases to arise (Glenk & Kothgassner, 2017).

Alongside these physical health benefits, we found that people with PTSD leave their home more often together with their assistance dog, make plans for their future and have an increased vitality. It has been shown that HAI can also have psychological effects, such as reduction of depression and anxiety. Beyond that, humans’ intrinsic motivation is activated via the involvement of animals, because due to biophilia (affinity to animals and nature, Wilson, 1984) people become emotionally involved when engaging in activities with animals (Wohlfarth, Mutschler, Beetz, & Schleider, 2014; Wohlfarth, Mutschler, Beetz, Kreuser, & Korsten-Reck, 2013). Furthermore, animals provide social support, which seems to be the most important variable in trauma recovery and is thought to improve healing processes (Tedeschi & Jenkins, 2019). According to the social support theory, animals provide direct social support by being a source of non-judgmental support and unconditional companionship, but also indirect support by facilitating human interactions for their owners (Tedeschi & Jenkins, 2019). The participants’ relationship with their dogs enables trust in people to some degree, leading to an improvement of their social relationships.

Almost all human beings have an inherent need to form and maintain a minimum quantity of interpersonal relationships, which is called the “need to belong” (Baumeister & Leary, 1995). While forming an attachment bond produces positive emotion in general, not doing so could lead to emotional distress (Baumeister & Leary, 1995). It is evident that psychological health problems are more common among people without social attachments (Baumeister & Leary, 1995). Arguably, it is helpful for PTSD patients to gain social contacts for their mental health, and subsequently this can partly improve their QoL. Since an attachment bond – characterized by providing feelings of safety and security, often in a mutually beneficial way – can also be formed between human and dog (Tedeschi & Jenkins, 2019), the addition of an assistance dog in patients’ lives can provide them with this necessary attachment.

An explanation of why it is easier for people with PTSD to get in touch with others when they have their assistance dog with them could be the sense of safety that the dog reportedly provides (through their company and assistance with tasks). Again, through the biophilia effect, a feeling of safety is provided by the presence of a calm and friendly animal leading to a reduction of psychological and physiological distress (Beetz, Schöfmann, Girgensohn, Braas, & Ernst, 2019).

Next to the elaborated positive effects of PTSD-assistance dogs on their handlers’ QoL, some burdens can also arise and must be taken into consideration. One point is the challenge of putting oneself into unpleasant situations for the purpose of training the dog. Even a puppy’s house training could challenge a PTSD-patient, if it for instance requires to go outside while it is dark, and their trauma has been triggered in the darkness before. By getting oneself into challenging situations, symptoms of reexperiencing the event, might be triggered. The risk of a decreased QoL from putting oneself in such a situation is a possible trade-off for increasing QoL once tough phases like puppyhood are concluded, for instance.

One more challenge concerning our target group that was raised within this study was the access and inclusion issues for both PTSD-assistance dogs and people with mental disabilities. In theory, a legal framework is provided by the Austrian federal disability act § 39a (2), which states that assistance dogs should be used for the purpose of expanding the self-determination and participation of people with disabilities in all areas of life. Therefore, people with disabilities who are accompanied by their assistance dog need free access to public places, buildings, and services (Federal Ministry of Labor, Social Affairs and Consumer Protection, 2015).

### Salivary cortisol measure

The results of the second part of our study are contrary to those of Bruckner (2019), and contrary to a meta-analysis performed by Cobb, Iskandarani, Chinchilli, and Dreschel (2016) which found no significant effects of dog type (e.g., assistance, companion, therapy, hunting) on salivary cortisol concentrations. Even though we used the same sampling materials, schedule and instructions for handlers as Bruckner (2019), the sample analysis was performed in the same institute using the equivalent EIA to carry out the process of data collection, and analysis was as similar as possible, this study found PTSD-assistance dogs to have lower, not higher, levels of salivary cortisol than companion and diabetic-signal dogs. A possible reason for this contrast of results might be seasonal variation or circadian effect in salivary cortisol concentrations, as was detected in humans (Persson et al., 2008). In addition, we could not completely control the participants’ handling of the samples.

We had to exclude one dog due to too low saliva volume.

Apart from reasons that cannot be completely ruled out due to variations in sampling, another reason for our unexpected results could be that dogs that have a close personal relationship with their handlers show lower salivary cortisol levels (Gunnar, 1998; Schöberl et al., 2012). The handler – dog attachment also has a significant effect on dogs’ stress levels (Schöberl et al., 2012). PTSD-assistance dogs continuously accompany their handlers, provide tactile stimulation and body contact (Lloyd, Johnston, & Lewis, 2019). Therefore, it is possible that an even closer bond between PTSD-assistance dog and handler is formed than it is the case for companion dogs, who usually do not accompany their handlers to the same extent. Additionally, it might be the case that companion dogs living in a family association form a relationship to one or several family members but may not have such a strong attachment to a single person. As stated by the participants of the current study, they see their assistance dogs as social partners, which matches the findings of Schöberl et al. (2012), for example, who found that the quality and strength of the bond between human and dog affects dogs’ salivary cortisol levels. Physical contact and touch increase the levels of the attachment hormone oxytocin (Nagasawa et al., 2015), leading to mental and physiological relaxation in humans with an even larger effect between humans and dogs with a close relationship (Beetz & Bales, 2016). The results of this study suggest that this effect may also be present vice versa, leading to lower cortisol concentrations in PTSD-assistance dogs as a result of social buffering. Oxytocin is secreted in response to stress, whereby secretion is enhanced by the presence and physical contact of a social partner, reducing the activity of stress response systems (Buttner, 2016).

Following up on the results of Bruckner (2019), who found lower cortisol values after dog walks but no differences between dogs’ stress and working stress, we also included PTSD-assistance dogs’ reported states before sampling. The finding of no significant differences between the states might be due to low feedback of the handlers regarding different occurrences in the dogs’ daily routines. However, other studies (Cobb, Iskandarani, Chinchilli, & Dreschel, 2016) did not find any differences in salivary cortisol levels after physical activity within one hour before sampling.

Further, we did not find a significant effect of time of day on cortisol levels, which is in line with the findings of Bruckner (2019) and other studies (e.g., Koyama, Omata, & Saito, 2003). Another factor that was controlled for within this study was age, since some studies (Goy-Thollot, Decosne-Junot, & Bonnet, 2007; Rothuizen et al., 1993) found an age-related increase in circulating cortisol in older dogs while others did not (Hennessy, Davis, Williams, Mellott, & Douglas, 1997; Mongillo, Prana, Gabai, Bertotto, & Marinelli, 2014; Reimers, Lawler, Sutaria, Correa, & Erb, 1990). Therefore, the effect of age on cortisol remains controversial. In the current study, the sample of PTSD-assistance dogs consisted of younger individuals on average than that of companion and alert dogs. However, the results do not show a significance between age and cortisol values. We also controlled for an effect of sex on salivary cortisol levels. Like in a study of Haubenhofer, Möstl, and Kirchengast (2005), no significant effect of sex was found.

### Limitations and future research

In the first part of this study, a qualitative approach was used since many relational aspects of HAI are inherently qualitative and difficult to obtain in quantitative methodologies (Tedeschi & Jenkins, 2019). Further studies would be useful to increase the generalizability of our results, with sampling being extended to other countries and more study participants.

A clear limitation is that a generalization of our results to other human groups accompanied by assistance dogs cannot be made. The findings of this study represent the personal experiences and opinions of PTSD-patients in Austria and Germany. Other disabled people with assistance dogs also have to deal with obstacles and discrimination in their everyday lives, which sometimes might be comparable to the findings of this study, but different groups and even individuals might have their own experiences.

Regarding the measurement of dogs’ stress levels, it is necessary to improve the sampling strategy for future studies to increase saliva volume, since low volume and empty samples are a major drawback in our study. The usage of an enriched, instead of a plain, swab (Meunier, Groessl, Reusch, Boretti, & Sieber-Ruckstuhl, 2021), for example, could be one possibility. In addition, another strategy to gain more samples to measure cortisol would be to use fecal (Schatz & Palme, 2001) or hair samples (Bryan, Adams, Invik, Wynne-Edwards, & Smits, 2013; van Houtert, Endenburg, Vermetten, & Rodenburg, 2022), which should be easier to collect for nonprofessionals. Moreover, physiological parameters as well as dogs’ behavior could be used to better understand whether dogs experience distress during or after different states, like working situations or dog walks.

## Conclusion

In conclusion, this study has provided evidence for PTSD-assistance dogs improving their handlers’ QoL by performing several assistance tasks and by being their social partner. However, having an assistance dog by one’s side also brings new challenges in everyday life that need to be considered. Two major problems are access issues for assistance dogs, and how people should behave when meeting a working assistance dog. Such issues must be treated in the future with special information campaigns, like advertisements or posters. Further, our study has provided valuable data for addressing the welfare of assistance dogs. PTSD-assistance dogs’ salivary cortisol levels are lower than those of the control groups, which could be explained by the dogs forming a very close attachment bond with their humans and being a social buffer for them.

## Supplementary Material

Supplementary material

## Figures and Tables

**Table 1:** Group membership, sex, age and breed of the subjects.

Group membership	Sex	Age	Breed
PTSD-assistance dog	M	2	Labrador Retriever
PTSD-assistance dog	M	1	Labrador Retriever
PTSD-assistance dog	M	2	Mixed breed
PTSD-assistance dog	F	3	Cavalier King Charles Spaniel
PTSD-assistance dog	F	4	Australian Shepherd
PTSD-assistance dog	F	7	Labrador Retriever
PTSD-assistance dog	F	NA	Labrador Retriever
PTSD-assistance dog	M	NA	Labrador Retriever
PTSD-assistance dog	M	10	Mixed Breed
Diabetic-signal dog	M	4	Labrador Retriever
Diabetic-signal dog	M	5	Retriever mix
Diabetic-signal dog	M	3	Labrador Retriever
Diabetic-signal dog	F	3	Labrador Retriever
Diabetic-signal dog	M	3	Retriever
Diabetic-signal dog	M	3	Labrador Retriever
Diabetic-signal dog	F	3	Labrador Retriever
Diabetic-signal dog	F	3	Labrador Retriever
Diabetic-signal dog	F	5	Labrador Retriever
Companion dog	F	5	Golden Retriever
Companion dog	F	3	Labrador Retriever mix
Companion dog	F	3	Golden Retriever
Companion dog	F	7	Golden Retriever
Companion dog	F	6	Mixed breed
Companion dog	F	5	Labrador Retriever mix
Companion dog	M	3	Golden Retriever
Companion dog	F	3	Labrador Retriever
