# Supplementary material for: A pilot study into the effects of PTSD-assistance dogs’ work on their salivary cortisol levels and their handlers’ Quality of life
Source: J Appl Anim Welf Sci. Author manuscript; Available in PMC 2025 Apr 1. (PMC7616523; doi:10.1080/10888705.2023.2259795)
Supplement: Supplementary material [file EMS197386-supplement-Supplementary_material.docx]

**Supplementary material from “A pilot study into the effects of PTSD-assistance dogs’ work on their salivary cortisol levels and their handlers’ Quality of Life”**

Karoline Gerwisch, Karl Weissenbacher, Michelle Proyer, Ludwig Huber

**S1 Qualitative online questionnaire**
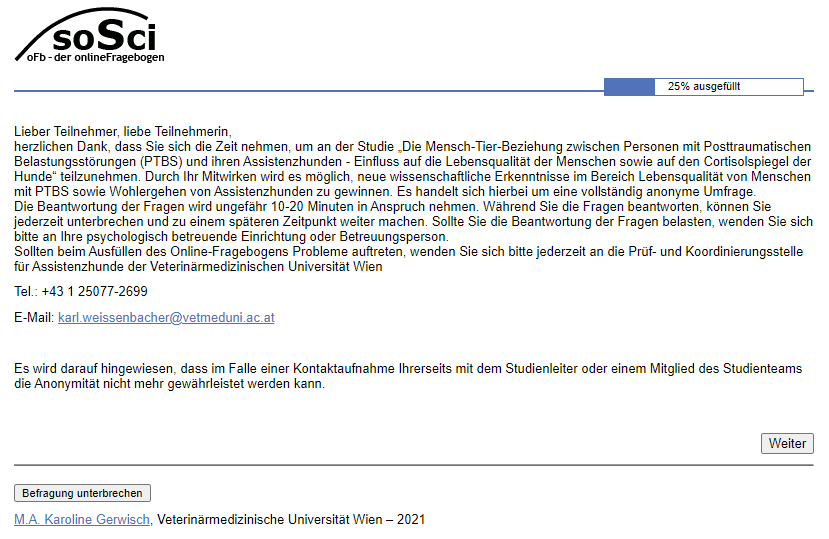

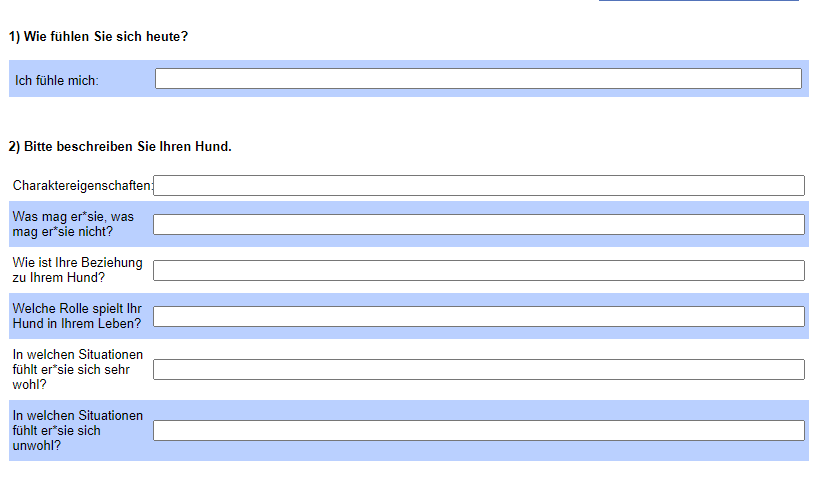

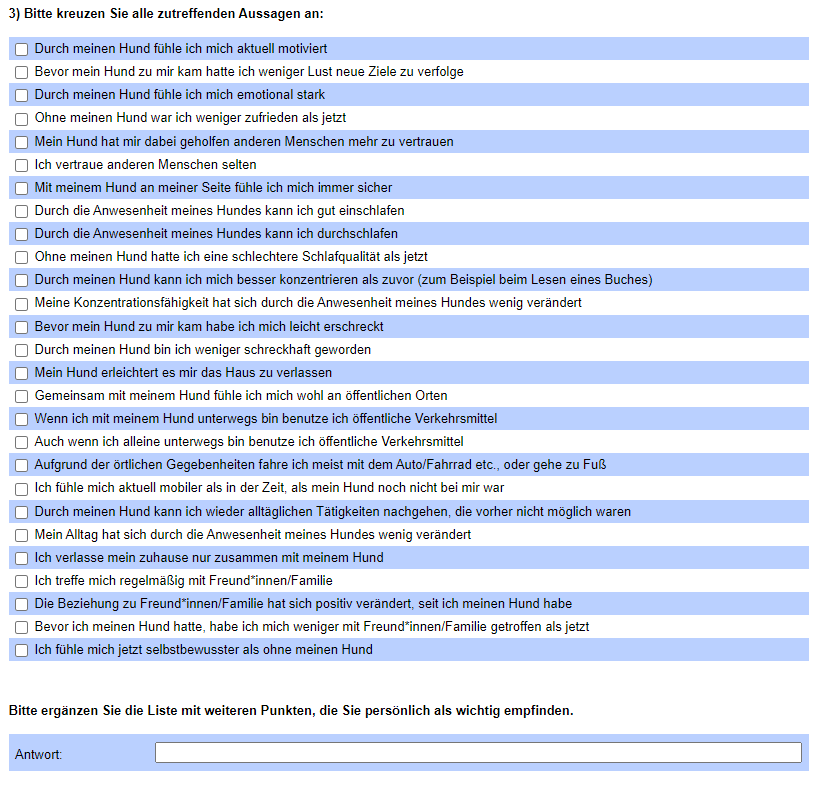

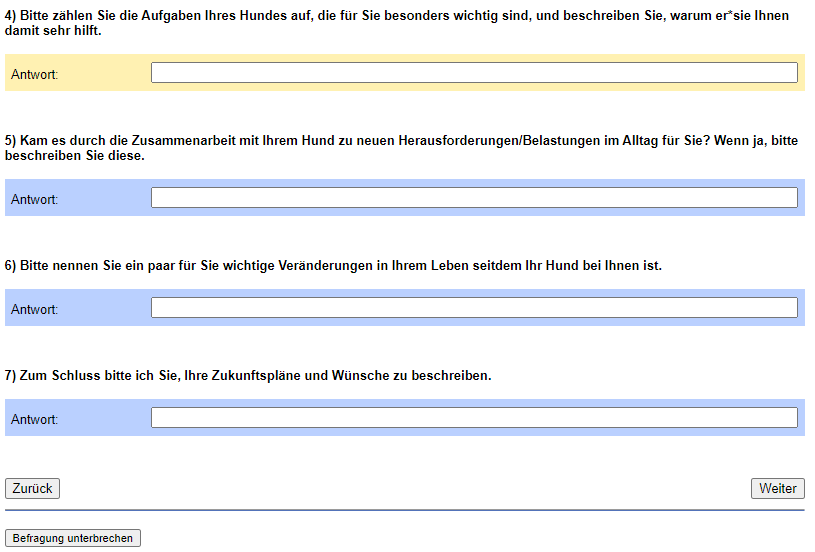

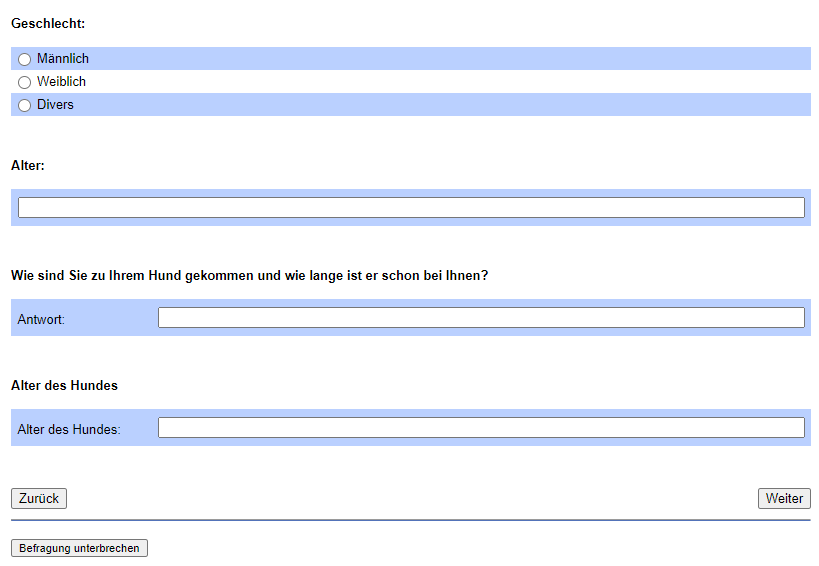


**S2 Salivary cortisol measure**

The dog handlers were asked not to feed the dog for a period of one hour before sampling, as this would contaminate the sample (SARSTEDT AG & Co.) which would affect the cortisol measures (Ligout et al., 2010). Participants were requested to wear disposable gloves to further prevent any contamination of the sample. The Salivette remained in the dogs’ cheek pouch for at least 60 seconds, but not longer than three minutes, since this could also affect the cortisol value (Kobelt et al., 2003). During collection, the dogs were praised and petted, and treats were only given after the Salivette was removed. The saliva soaked Salivette was returned to its tube, labelled, and frozen. Samples were then transported in Styrofoam boxes with freezer packs to the Institute of Biochemistry of the University of Veterinary Medicine Vienna for analysis.

Further, participants were asked to take the samples when the dogs were in a stage of rest. This could also be during a working task, e.g., cuddling on the sofa with the handler to provide comfort. The reason was to avoid sampling immediately after stressful situations since this would bias the results. Participants noted the times of sampling on the Salivette and on a protocol. Any extraordinary occurrences which happened before sampling were also noted, in order to test their effect on the dog' distress. Extraordinary occurrences were categorized into company work (accompanying the handler to different places e.g., stores, public transport, social events), stress (possibly stressful events not connected to assistance work e.g., thunderstorms), walk (dog was walked before), and working stress (possibly stressful events related to assistance dog work e.g., aiding when handler had a seizure).

Samples were centrifuged for ten minutes at 3750 rpm to extract the saliva. Fifty μl saliva diluted 1:10 were used for the measurement of the cortisol value.

The samples of companion dogs and signal dogs for diabetic people were taken in 2017 using the same kind of cotton roller (Cortisol-Salivette®, SARSTEDT AG&Co, Germany). Instructions for each of the participants were identical, and storing, transport and analysis of the samples was performed always in the same manner.

**S3 Statistical Analysis**

To avoid pseudoreplication we also included subject ID as well as a combination of subject and date (subj.date) to control for any day-to-day variation. To avoid overconfident models and to keep type I error rate at the nominal 5% level, we also included all theoretical identifiable random slope components (Barr et al., 2013; Schielzeth & Forstmeier, 2009).

After fitting the model, we checked whether the assumptions of normal distribution and homogeneous residuals were fulfilled by visual inspection of a QQ-plot of residuals and residuals plotted against fitted values. These indicated no major deviations from these assumptions. We verified absence of collinearity by calculating the Variance Inflation Factor (VIF) using the R package “car” version 3.0-12 (Fox & Weisberg, 2019). This revealed that collinearity was not an issue (all VIFs <1.7). We visually inspected the best linear unbiased predictors (BLUPs) per level of the random effects, which were approximately normally distributed (Harrison et al., 2018).

We assessed model stability with regard to the model estimates by comparing the estimates from the model, including all data with estimates obtained from models in which the levels of random effects were excluded one at a time (Nieuwenhuis et al., 2012). This revealed the model to be of good stability with respect to both fixed effects and random effects.

**S4 Results**

***QoL measure***


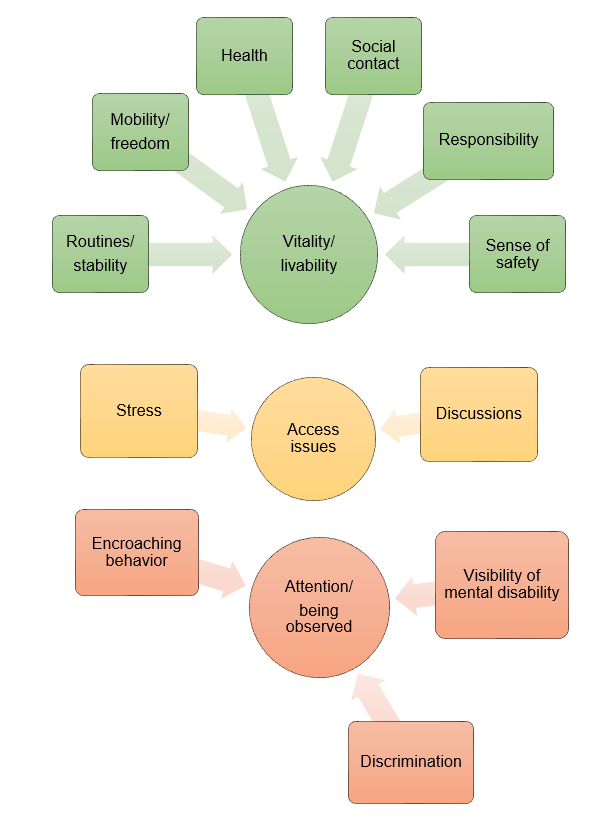


***Figure S1.*** *Result categories: “Vitality/livability” describes the current state of the participants and how their assistance dogs help them to gain a kind of lust for life and to cope with daily challenges. “Access issues” and “Attention/being observed” take up the more challenging sides of having a PTSD-assistance dog and clarify that new obstacles can arise.*

In general, the results of our study suggest that PTSD-assistance dogs contribute to their handlers’ QoL by increasing their vitality. However, there are also more demanding sides of working with a PTSD-assistance dog, and our results suggest that new obstacles indeed arise. The following points summarize the improvements of participants’ QoL as well as their experienced challenges (see Fig. 1).

*Health*
Because of the dogs’ assistance work, study participants stated to suffer less from dissociations and seizures. During such episodes, patients were at a higher risk of injuries, for instance, by falling or violence of passerby/health personnel. The reduced risk from the dogs’ assistance additionally decreased a need for surgery and hospitalizations. Self-harmful behavior and eating disorders could be improved by the signaling and disrupting of assistance dogs.

Regarding the subjective experiences of more than half of the participants, having a PTSD-assistance dog can help falling asleep and provide a better quality of sleep compared to the sleeping quality before having a dog.

*Routines and stability*Our results also indicated that participants had improved routines and stability in their lives because of their dogs. Daily tasks like walking and feeding the dog had to be done, and PTSD-patients did not perceive whole days as threatening anymore, but rather days were separated into manageable segments.

*Responsibility*
Having routines was interwoven with the handlers’ responsibility for their assistance dogs, which also drew their attachment to their dog closer, making them able to experience some form of love. Since people with PTSD might suffer from numbing of emotions, a flattening of general psychological responsiveness or feeling alienated from others, their assistance dogs as main attachment figures could help them to overcome this emotional state. Apart from that, another reported outcome was that with responsibility comes burden, as respondents found themselves in challenging, frightening, or even unpleasant situations. Examples given were the socialization process of puppies when they need to associate situations positively, which the handler herself is afraid of, or having to go outside at night in the phase of housetraining. Putting themselves in such situations was very challenging for participants, particularly when they had experienced their trauma at night in the dark.

*Social contact*Our results pointed toward assistance dogs being an attachment figure whom patients formed a close bond with, and patients were able to establish and maintain their social contacts and friendships. Moreover, half of the sample stated that their relationships with family and friends changed for the better since having their dog.

*Sense of safety*

The assistance work of the dogs, such as blocking strangers while waiting in line at a store, and the mere company of the dogs provided their handlers with a sense of safety. Before having a dog, the respondents had difficulties leaving their homes and following daily schedules. An assistance dog that enabled safety even helped respondents to attend appointments on their own or use public transport.

*Mobility and freedom*

Following up on the previous point, PTSD-assistance dogs gave their handlers higher mobility and freedom in their day-to-day lives.

*Attention*

Another finding of our study was that participants reported experiencing the encroaching behavior of strange people when they were out with their dogs. Patients with an assistance dog got more attention than without, and were more observed by others. They mentioned that people took videos of their working dog, wanted to pet them, and asked unpleasant questions about the dogs or patients’ “invisible” disability, which those affected perceived as discriminating. Their disability became more noticeable through their assistance-dog while they preferred to be left alone and rather not interact with others.

*Access issues*

People with (mental) disabilities face entry refusal at public institutions and must fight for their right to access with their assistance dog. The ones affected described it as very stressful to discuss and justify why they must go into a store, to an appointment, etc. accompanied by a dog. It was also described that the stress of arguing with people who are unaware of assistance dogs’ rights was the biggest burden of having such a dog. Despite the facilitation brought by a PTSD-assistance dog, they also brought new challenges and burdens.

**References**

Barr, D. J., Levy, R., Scheepers, C., & Tily, H. J. (2013). Random effects structure for confirmatory hypothesis testing: Keep it maximal. *Journal of Memory and Language*, *68*(3), 255–278.

Fox, & Weisberg. (2019). *Applied Regression 3E*. https://socialsciences.mcmaster.ca/jfox/Books/Companion/ (accessed 03.05.2023)

Harrison, X. A., Donaldson, L., Correa-Cano, M. E., Evans, J., Fisher, D. N., Goodwin, C. E. D., Robinson, B. S., Hodgson, D. J., & Inger, R. (2018). A brief introduction to mixed effects modelling and multi-model inference in ecology. *PeerJ*, *6*, e4794.

Ligout, S., Wright, H., Driel, K. van, Gladwell, F., Mills, D. S., & Cooper, J. J. (2010). Reliability of salivary cortisol measures in dogs in training context. *Journal of Veterinary Behavior*, *5*(1), 49.

Nieuwenhuis, R., Grotenhuis, M. te, & Pelzer, B. (2012). influence.ME: Tools for Detecting Influential Data in Mixed Effects Models. *The R Journal*, *4*(2), 38–47.

Schielzeth, H., & Forstmeier, W. (2009). Conclusions beyond support: Overconfident estimates in mixed models. *Behavioral Ecology*, *20*(2), 416–420.
